# OpenAI o1-Preview vs. ChatGPT in Healthcare: A New Frontier in Medical AI Reasoning

**DOI:** 10.7759/cureus.70640

**Published:** 2024-10-01

**Authors:** Mohamad-Hani Temsah, Amr Jamal, Khalid Alhasan, Abdulkarim A Temsah, Khalid H Malki

**Affiliations:** 1 Pediatric Intensive Care Unit, Pediatric Department, College of Medicine, King Saud University Medical City, King Saud University, Riyadh, SAU; 2 Family and Community Medicine Department, King Saud University, Riyadh, SAU; 3 Pediatric Nephrology Department, King Saud University, Riyadh, SAU; 4 Dental Department, Specialized Medical Center Hospital, Riyadh, SAU; 5 College of Medicine, King Saud University, Riyadh, SAU

**Keywords:** transparency in ai and llms black box, chain of thought, hallucinations, ai ethics, genetic disease discovery, complex reasoning, healthcare applications, artificial intelligence, chatgpt, openai o1-preview

## Abstract

This editorial explores the recent advancements in generative artificial intelligence with the newly-released OpenAI o1-Preview, comparing its capabilities to the traditional ChatGPT (GPT-4) model, particularly in the context of healthcare. While ChatGPT has shown many applications for general medical advice and patient interactions, OpenAI o1-Preview introduces new features with advanced reasoning skills using a *chain of thought* processes that could enable users to tackle more complex medical queries such as genetic disease discovery, multi-system or complex disease care, and medical research support. The article explores some of the new model’s potential and other aspects that may affect its usage, like slower response times due to its extensive reasoning approach yet highlights its potential for reducing hallucinations and offering more accurate outputs for complex medical problems. Ethical challenges, data diversity, access equity, and transparency are also discussed, identifying key areas for future research, including optimizing the use of both models in tandem for healthcare applications. The editorial concludes by advocating for collaborative exploration of all large language models (LLMs), including the novel *OpenAI o1-Preview*, to fully utilize their transformative potential in medicine and healthcare delivery. This model, with its advanced reasoning capabilities, presents an opportunity to empower healthcare professionals, policymakers, and computer scientists to work together in transforming patient care, accelerating medical research, and enhancing healthcare outcomes. By optimizing the use of several LLM models in tandem, healthcare systems may enhance efficiency and precision, as well as mitigate previous LLM challenges, such as ethical concerns, access disparities, and technical limitations, steering to a new era of artificial intelligence (AI)-driven healthcare.

## Editorial

**Figure 1:**
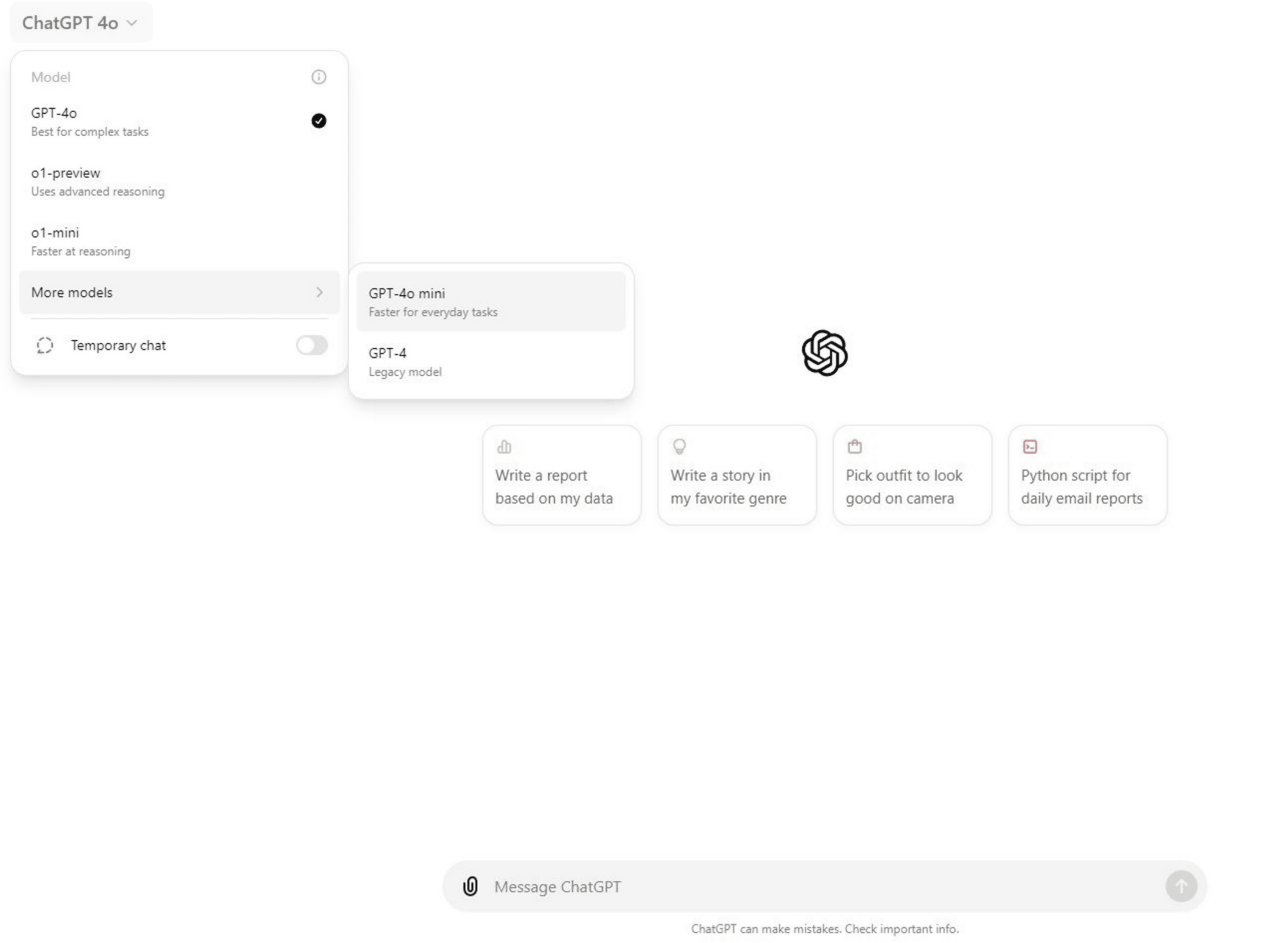
Screenshot taken on September 14, 2024, from the desktop version of the ChatGPT interface, displaying the available models under the GPT-4o and o1-Preview families. The image shows a comparison of models, including GPT-4o (optimized for complex tasks), the o1-Preview model (designed for advanced reasoning), and the o1-mini model (optimized for faster reasoning). This illustrates the increasingly available options for users to select different AI capabilities, depending on the complexity and nature of the tasks they intend to perform.

In another advancement for generative artificial intelligence (AI), OpenAI released its latest model, the OpenAI o1-Preview, on September 12, 2024 [1]. This model leads to a new era of AI-powered reasoning, outpacing traditional models like ChatGPT (GPT-4o) in handling complex, logic-heavy tasks (Figure 1). For the healthcare sector, this presents exciting potential. While ChatGPT has impacted patient engagement, medical education, and research support, the o1-Preview model promises to elevate AI’s capabilities in tackling deeper, more intricate medical queries. For example, the o1-Preview model could excel in providing differential diagnoses for rare conditions based on subtle symptomatology, generating treatment plans that incorporate a wide range of comorbidities, or navigating complex genomic data to identify potential genetic markers for personalized medicine. However, this new frontier of AI model in medical reasoning could introduce novel challenges, such as the transparency and interpretability of AI outputs, which deserve careful attention [2,3].

OpenAI o1-Preview: Designed for complex reasoning

Unlike ChatGPT, which tends to provide instant answers without much internal deliberation, the OpenAI o1-Preview is built to "think" step-by-step using a technique called "chain of thought reasoning [1]." This method allows the model to break down complex problems, mimicking how humans approach difficult tasks. As demonstrated in performance tests, o1-Preview has significantly outperformed previous models, particularly in fields requiring deep logical reasoning like mathematics and the sciences, including biology and chemistry​ [1]. This is particularly crucial for healthcare applications where accurate diagnosis, treatment strategies, and research rely on profound, multidisciplinary knowledge.

Moreover, its high-level reasoning could provide better support for interdisciplinary teams tackling complex, multi-system diseases, like lupus, which involve several interactions between different body systems [4]. The model could potentially analyze diverse data points from various specialties-rheumatology, nephrology, and immunology, for example, offer integrated insights into disease progression, potential treatment responses, and patient management. Similarly, for conditions like cancer, where treatment requires a multifaceted approach involving oncology, surgery, and radiology, o1-Preview could assist in optimizing care pathways, ensuring that personalized treatment plans consider every angle, from genomic mutations to predicted patient outcomes. By supporting such intricate clinical decision-making, o1-Preview may improve care quality, enhance multidisciplinary collaboration, and advance precision medicine [5,6].

Healthcare opportunities with OpenAI o1-Preview

The enhanced reasoning capabilities of the o1-Preview model make it uniquely suited for more advanced tasks in healthcare, particularly where intricate logical problem-solving and data interpretation are required (Table 1) [1]. One such area is genomic and genetic disease discovery, where o1-Preview could assist in mining vast genomic datasets to identify correlations, mutations, or patterns that signify rare diseases or novel therapeutic targets. Traditional approaches to analyzing genetic data may fall short due to the complexity of the information involved. However, with o1-Preview’s chain-of-thought reasoning, healthcare professionals could unlock diagnostic insights that were previously unattainable, paving the way for earlier detection and more targeted interventions for rare conditions.

**Table 1:** Comparative analysis of ChatGPT (GPT-4) vs. OpenAI o1-Preview in healthcare applications

Feature	ChatGPT (GPT-4)	OpenAI o1-Preview	Possible future research
Reasoning capabilities	Basic logical reasoning, struggles with complex tasks	Advanced, step-by-step reasoning (chain of thought)	How does step-by-step reasoning enhance diagnostic accuracy?
Response time	Fast	Slower due to complex reasoning	Can models be optimized for faster yet accurate responses in healthcare settings?
Medical application	Good for patient communication, general medical advice	Ideal for complex medical reasoning, genetic analysis, and research tasks	Which medical specialties benefit most from each model?
Hallucination risk	Moderate, can hallucinate in complex scenarios	Reduced in complex scenarios but still possible	How can hallucinations be minimized further in real-world clinical applications?
Dataset diversity	Diverse, but not guaranteed to represent all global populations	Requires validation to ensure diverse representation	Can datasets be expanded to include more diverse, underrepresented populations?
Equity of access	Widely accessible, free version available	Limited to premium users (currently)	How can access to advanced models be improved globally?
Communication skills	Excellent for human-like conversation	Weaker for general conversation, focuses more on logic-heavy tasks	How to improve conversational abilities for medical professionals and patient interaction?
Ethical Considerations	Black box decisions, limited transparency	The black box issue partially addressed by chain of thought, but still a concern	How to further improve transparency in AI decision-making?
Training level	Trained to provide broad but surface-level knowledge	PhD-level reasoning in physics, chemistry, biology	Which areas of medicine require even more specialized AI training?

Another promising area where o1-Preview could drive innovation is in medical research and drug discovery [7,8]. By breaking down complex biological processes and analyzing drug interactions, the model could aid researchers in generating hypotheses, designing studies, and predicting the outcomes of experimental treatments. Its Ph.D.-level understanding of disciplines such as biology and chemistry allows it to simulate and analyze complex mechanisms in ways that are traditionally labor-intensive and time-consuming. This could lead to faster drug development and improved therapeutic options for patients suffering from diseases that currently lack effective treatments, though further research is needed.

In complex patient care management, o1-Preview offers the potential to transform how clinicians manage chronic, multi-system diseases. For conditions such as diabetes or cardiovascular disease, the model can evaluate intricate care pathways by considering various patient-specific factors such as genetic background, lifestyle, and co-morbidities. The result is a more personalized, precise, and effective treatment plan, improving patient outcomes and reducing the risk of complications. This model may act as a virtual consultant, providing detailed insights that would be difficult for any single healthcare provider to gather [9,10].

In medical education, o1-Preview could serve as a powerful learning tool for both students and professionals. Its ability to handle intricate logical problems and deliver reasoning-based explanations makes it particularly well-suited for training in complex fields such as surgery or radiology. Through simulated scenarios, students can learn how to navigate difficult clinical decisions, while professionals can use the model as a resource for staying updated on the latest medical advancements. In this way, the o1-Preview could contribute significantly to the future of medical training by offering a more interactive and intellectually stimulating learning environment.

A key opportunity lies in the collaborative use of ChatGPT and o1-Preview, where each model’s strengths can complement the other. While o1-Preview excels in deep reasoning and complex problem-solving, ChatGPT continues to outperform in communication, making it ideal for patient interactions, telemedicine, and documentation tasks. This combination of models could revolutionize healthcare delivery. For instance, ChatGPT could be employed for communicating diagnoses or treatment plans in a patient-friendly manner, while o1-Preview works behind the scenes, performing rigorous medical reasoning and analysis. The integration of these models could fill current gaps in healthcare, ensuring that both the precision of care and the human touch remain intact. According to recent studies, using models like ChatGPT in patient communication could improve patient engagement and adherence to medical advice, highlighting the importance of effective communication in medical practice.

Challenges and ethical considerations

As promising as the o1-Preview model is, it introduces a series of challenges that require immediate attention from the healthcare community. One significant concern is its slower response time. Unlike ChatGPT, which provides rapid responses, o1-Preview takes longer due to its step-by-step reasoning process. While this approach enhances the model's capacity for handling complex queries, in time-sensitive healthcare environments, particularly acute care settings, these delays could present a problem. Solutions such as hybrid models that delegate simpler tasks to faster systems, while reserving o1-Preview for more complex reasoning, may be necessary to mitigate this issue.

Another challenge involves equity in access to advanced AI. Currently, o1-Preview is available only to premium ChatGPT users, which raises concerns about widening the digital divide in healthcare. The limited availability of this advanced tool could exacerbate existing health disparities by restricting access to those who can afford premium models. As AI continues to evolve, ensuring equitable access to these technologies will be crucial in preventing further gaps in healthcare outcomes.

Ethical implications surrounding AI-driven decision-making also come to the forefront with the o1-Preview. As the model engages in more sophisticated reasoning, the transparency of its decision-making process becomes critical. The longstanding *black box* issue in large language models (LLMs), where the internal logic of AI outputs remains a concern, persists even with o1-Preview’s chain-of-thought reasoning. Although this method allows for greater insight into how the model arrives at its conclusions, the underlying complexities still pose challenges for healthcare professionals who need to trust and verify the AI’s recommendations. More research is needed to better understand how transparent and interpretable the model’s reasoning truly is.

Another consideration is the diversity of training data. Like its predecessors, o1-Preview depends heavily on the diversity of the data it is trained on. To avoid perpetuating biases in diagnosis and treatment, the model’s training data must include a broad range of healthcare scenarios from across different populations. Failing to do so risks the AI providing biased recommendations that could harm underrepresented groups, a concern that warrants further exploration and validation.

Finally, the issue of hallucinations where AI generates inaccurate or irrelevant content remains, albeit with improvements [11,12]. While o1-Preview has shown a reduction in hallucinations, especially in complex queries, it is not entirely immune to them. In medical contexts, such inaccuracies could have serious, if not life-threatening, consequences. This underscores the need for ongoing research and real-world validation to ensure the model's accuracy and reliability before it can be trusted in critical healthcare decisions.

Need for further research and exploration.

Despite the immense promise of o1-Preview, further research is essential to fully understand its value in specific medical fields. Comparative studies that evaluate o1-Preview and ChatGPT across diverse healthcare applications such as diagnostics, treatment recommendations, and patient education are urgently needed. Understanding which model excels in specific contexts will be key to optimizing their use in practice.

Moreover, there is a compelling need to explore hybrid systems that combine the strengths of both models. ChatGPT’s speed and proficiency in communication, particularly for patient interactions and documentation, could complement o1-Preview's deep reasoning capabilities. As an initial version, o1-Preview currently lacks several features that contribute to ChatGPT's usefulness, such as web browsing capabilities and the ability to upload files and images [1]. For many routine tasks, GPT-4o is likely to remain more effective in the short term. Therefore, such systems could streamline healthcare workflows, with ChatGPT handling patient-facing tasks while o1-Preview operates in the background, solving complex medical problems. This layered approach holds enhanced potential for improving the quality of clinical decision-making, but it must be thoroughly evaluated for safety, reliability, and accessibility.

Conclusions

The release of OpenAI o1-Preview marks a new landmark advancement in the potential application of AI and advanced LLMs to healthcare. Its advanced reasoning abilities may revolutionize how healthcare professionals tackle complex medical queries, from identifying unknown diseases to developing personalized care plans. However, its slower processing speed, potential barriers to access, and possible ethical challenges require a balanced approach. The healthcare community should proceed with both optimism and caution, ensuring that this technology is integrated carefully and responsibly into medical practice. The future of AI in healthcare is more intricate, thoughtful, and promising than ever before. By combining rigorous research with innovative integration strategies, o1-Preview and other LLM models have the potential to transform patient care and outcomes. The path forward is becoming clearer: through collaboration and continued exploration, healthcare professionals, researchers, and technologists can unlock more potentials of AI to create a more efficient, equitable, and intelligent healthcare system. Together, we stand at the threshold of an exciting new era; let's explore it.
